# Is Recovery from Cannabis Dependence Possible? Factors that Help or Hinder Recovery in a National Sample of Canadians with a History of Cannabis Dependence

**DOI:** 10.1155/2020/9618398

**Published:** 2020-04-15

**Authors:** Esme Fuller-Thomson, Janany Jayanthikumar, Melissa L. Redmond, Senyo Agbeyaka

**Affiliations:** ^1^Professor & Sandra Rotman Endowed Chair, Director of the Institute for Life Course & Aging, Factor-Inwentash Faculty of Social Work, Cross-Appointed to the Department of Family & Community Medicine and the Faculty of Nursing, University of Toronto, 246 Bloor St. W., Toronto, ON M5S 1V4, Canada; ^2^Factor-Inwentash Faculty of Social Work, University of Toronto, 246 Bloor St. W., Toronto, ON M5S 1V4, Canada; ^3^Assistant Professor, School of Social Work, Carleton University, 1125 Colonel By Drive, Ottawa, ON K1S 5B6, Canada

## Abstract

**Objectives:**

To identify among Canadian adults who have ever been dependent upon cannabis, the prevalence of risk and protective factors associated with (1) cannabis remission, (2) the absence of psychiatric disorders or addictions in the past year (APD), and (3) positive mental health (PMH).

**Method:**

Data from Statistics Canada's nationally representative 2012 Canadian Community Health Survey-Mental Health (*n* = 20, 777, of whom 336 have a history of cannabis dependence) was used. Chi-square tests and logistic regression analyses were conducted. The World Health Organization Composite International Diagnostic Interview (WHO-CIDI) measures were used to determine lifetime cannabis dependence, past-year remission from cannabis depression, and the absence of psychiatric disorders in the past year (APD) (i.e., no suicidal ideation, depressive episodes, anxiety disorders, bipolar disorders, or any substance dependence). PMH is comprised of three factors: APD, happiness or life satisfaction and social and psychological well-being.

**Results:**

Among those with a history of cannabis dependence, 72% were in remission from cannabis dependence. Although 53% were free of major psychiatric disorders and any substance dependence and 43% of respondents were in PMH, these percentages were dramatically lower than those without a history of cannabis dependence (92% and 74%, respectively). Positive outcomes were more common among women, older respondents, those with higher levels of social support, and those who had never had major depressive disorder or generalized anxiety disorder.

**Conclusion:**

Although many Canadians with a history of cannabis dependence achieve remission and a large minority are truly resilient and achieve PMH, many are failing to thrive. Targeted outreach is warranted for the most vulnerable individuals with a history of cannabis dependence (e.g., men, younger respondents, those with low social support and a history of mental illness).

## 1. Introduction

Cannabis is the most widely consumed illicit drug in the USA, [1] and Canada [2]. Between 2002 and 2013, there was a doubling of the percentage of Americans who had used cannabis in the preceding year [3]. Nearly three out of every ten users of cannabis have a cannabis use disorder (CUD), defined by abuse and/or dependence on cannabis [3]. Nationally representative US data from 2012-2013 indicate that 2.9% of American adults had a diagnosis of cannabis use disorder, which represented 31% of current users of cannabis [3]. An estimated 14.76 million Americans have ever abused and/or been dependent upon cannabis [4]. Canadian data from 2012 concluded that approximately 12% of Canadians have used cannabis in the past year and 1.3% of individuals aged 15 and older have ever been cannabis dependent during their lifetime [5].

Cannabis use disorder has been associated with numerous detrimental outcomes and behaviors including lower educational and occupational achievement [6, 7], psychiatric symptomology [8, 9], neuropsychological deterioration [10], impaired driving [11], higher risk of serious injury or fatality in motor vehicle crash [12, 13] and increased morbidity [2]. 

Despite the myriad of negative consequences associated with cannabis dependence, many individuals who are cannabis dependent experience remission, with a recent systematic review suggesting one in six individuals with cannabis dependence achieve remission annually [14]. Half of those with cannabis dependence remit within 6 years of dependence onset and the lifetime probability of cannabis remission is an encouraging 97.2% [15]. This compares favourably with lifetime remission rates from dependence of 83.7% for nicotine and 90.6% for alcohol [15].

A greater understanding of the factors associated with remission is crucial for timely and effective treatment and prevention interventions [15]. Several characteristics make it less likely for individuals to remit from cannabis dependence including male gender, dependence on additional substances, and a history of mental illness [15]. The likelihood of remission increases with the number of years since the original onset of dependence [15]. In Canada, the average age of initiation of cannabis use is 18.7 years of age [16]. In addition to the benefits of having more years, on average, in which to remit since they became dependent, older individuals are thought to be more likely to remit due to decreased impulsivity with age, more concerns about the impact of drug use on health problems and greater awareness of the social and legal consequences of substance abuse [17, 18, 19]. Additionally, those with a history of cannabis dependence who are employed are more likely to be in remission than those who are unemployed [20–22]. Income level was not significantly associated with remission [15]. 

Almost all those with a history of cannabis dependence in one national US study also had a diagnosis of another psychiatric disorder at some point in their lives (99%) including 57% with mood disorders, and 56% with anxiety disorders, [15]. Neither mood nor anxiety disorders were associated with remission [15]. Childhood adverse experiences, such as exposure to chronic parental domestic violence; childhood physical or sexual abuse, are associated with a higher prevalence of substance dependence [23] and a lower prevalence of PMH [24].

We are interested in three stages of recovery: (1) Remission from substance use disorder occurs when individuals no longer meet diagnostic criteria for substance use disorder over the span of a year. There are multiple definitions for “recovery from substance dependence” [25] but most include conditions of psychosocial functioning, beyond mere abstinence. (2) After achieving remission from cannabis dependence, the next stage in recovery is achieving freedom from any substance dependence or psychiatric disorder in the past year (Absence of Psychiatric Disorders, APD). (3) The ultimate stage in recovery goes beyond remission and the absence of psychiatric disorder, including a state of psychological health, wellness, and quality of life [25, 26]. Positive mental health (PMH) as a concept incorporates other domains of an individual's functioning and wellbeing as outcomes beyond remission. Keyes [26] conceptualizes PMH (also known as complete mental health) as a state based on the following: (1) the absence of mental illness in the last year (i.e., no serious suicidal thoughts, drug or alcohol abuse, and mental illness—APD, described above), (2) experiencing daily or almost daily happiness or life satisfaction in the last month, and (3) positive social and psychological well-being.

Very few studies, and none with national Canadian data (to our knowledge) have identified negative and positive correlates of remission from cannabis dependence. Furthermore, no studies that we are aware of have looked at correlates in this population of the more complete measures of recovery (APD & PMH).

Understanding the predictors of cannabis remission and full recovery to optimal well-being plays a critical role in the development and implementation of effective interventions which promote remission [21]. Identifying predictors that are most likely to support recovery allows clinicians to better target and treat individuals experiencing cannabis dependence.

The objectives of this study are to identify, in a nationally representative sample of Canadians, the extent to which a history of cannabis dependence is associated with (1) any addictions or psychiatric disorder in the past year (APD), and (2) positive mental health (PMH). Furthermore, among Canadian adults who have ever been dependent upon cannabis, to identify the prevalence of, and socio-demographic factors, mental health characteristics, and history of adverse childhood experiences associated with (1) cannabis remission, (2) APD, and (3) PMH.

## 2. Methods

### 2.1. Sample

As has been discussed elsewhere [27–29] secondary analyses were conducted using the nationally representative 2012 Canadian Community Health Survey-Mental Health (CCHS-MH) a public data set [30]. The 2012 CCHS-MH has an overall response rate of 68.9% [30]. This study utilized two sub-samples from the CCHS-MH study population. First, all participants with complete data on questions about lifetime cannabis dependence, past-year cannabis dependence, past-year mental illness and suicidality and past year positive mental health and covariates were included in this analysis (*n* = 20, 777). Questions concerning adverse childhood experiences were only asked among those over the age of 20, hence, this current study excluded those under 20 years of age. The second sub-sample consisted of adults with a lifetime diagnosis of cannabis dependence and complete data on the relevant variables (*n* = 336).

### 2.2. Measures

#### 2.2.1. Outcome Variable

Three different outcome measures were evaluated; (a) freedom of cannabis dependence in the past-year was based on the reliable and valid World Health Organization version of the Composite International Diagnostic Interview (WHO-CIDI); (b) the absence of psychiatric disorders (APD) in the past year was based on no suicidal ideation in the past year nor depressive episode, anxiety disorders, bipolar disorders, nor alcohol or drug dependence including cannabis and other drugs. The World Health Organization's version of the Composite International Diagnostic Interview (WHO-CIDI), a structured diagnostic interview that generates diagnosis according to the Diagnostic and Statistical Manual of Mental Disorders, Fourth Edition (DSM-IV) and the International Classification of Disease (ICD-10) was used to derive these variables. Finally, (c) positive mental health (measured as a binary variable) consisted of three parts: (1) the absence of psychiatric disorders in the past year as was described above; (2) emotional well-being (i.e., life satisfaction or happiness) and; (3) social and psychological well-being. The latter two were assessed using the Mental Health Continuum - Short Form (MHC-SF) [31]. The MHC-SF examines positive mental health using 14-items that assesses psychological well-being (e.g., during the past month, how often did you feel that you liked most parts of your personality?), emotional well-being (e.g., during the past month, how often did you feel: happy and/or satisfied with your own life?), and social well-being (e.g., during the past month, how often did you feel that you had something important to contribute to society?) [30, 31]. The MHC-SF has well established psychometric properties [31]. Respondents were categorized as being in “positive mental health” if they stated at least 1 of the 2 measures of emotional well-being (i.e., happiness and/or life satisfaction in past year) and a minimum of 6 of the 11 measures of psychological and/or social well-being “every day” or “almost every day” during the past month in tandem with the absence of any of the above listed forms of mental illness in the past year. Additional information can be found at Statistics Canada [30]. Our version was slightly modified by removal of one-question from the original 14-item instrument. The original version had included the question “interested in life?” in addition to “happy” and “satisfied with life” in the “emotional well-being” category. We felt that it was possible to be interested in life without being in optimal mental health and therefore removed it from the measure which resulted in the instrument having only 13 items now.****In this study, the internal consistency (Cronbach's alpha) for the 13-items was high (0.89).

#### 2.2.2. Key Exposure Variable

*Lifetime Cannabis Dependence.* Respondents were categorized as having a lifetime cannabis dependence if they met the WHO-CIDI definition for Cannabis Dependence criteria which requires having at least three symptoms of cannabis dependence (tolerance, withdrawal, increased consumption, attempts to quit, time lost, activities reduced, and continued use)” in the same 12-month period. For additional information, please see Statistics Canada [30].

#### 2.2.3. Other Variables in the Analyses

*Socio-demographic variables* investigated included sex and race (Non-Aboriginal White versus non-White and/or Aboriginal, based on self-report), age (measured in decades), and annual household income. The sum of three adverse childhood experiences was assessed (range of responses from 0 to 3). Exposure to chronic parental domestic violence was a dichotomous variable that was established if a participant indicated that prior to age 16 they had heard or seen in their home at least 11 times their “parents, step-parents or guardians hit each other or another adult”. Participants who responded at least once to the following questions were categorized as experiencing childhood sexual abuse: “How many times did an adult force you or attempt to force you into any unwanted sexual activity, by threatening you, holding you down or hurting you in some way?” Childhood physical abuse was derived by asking respondents if “an adult had slapped them on the face, head or ears or hit or spanked them with something hard to hurt them at least three times and/or pushed, grabbed, shoved, or threw something at them to hurt them at least three times and/or an adult had at least once kicked, bit, punch, choked, burned, or physically attacked them”.

Social support factors were assessed by using marital status (married/common-law versus single/divorced/widowed); and the 10-item Social Provisions Scale which ranged from 10 to 40 with higher scores indicating higher perceived levels of social support. The following forms of social provision were investigated in the scale: Attachment, Guidance, Social Integration, Reliable Alliance, and Reassurance of Worth. For more information please see Statistics Canada [30].

Lifetime generalized anxiety disorder and lifetime major depressive disorders were assessed using the WHO-CIDI lifetime criteria for each disorder. Further details about these measures are available [30].

### 2.3. Statistical Analysis

In the full sample (*n* = 20, 777), the characteristics of those with and without a history of cannabis dependence were compared using chi-square tests for categorical variables and independent *t*-tests for continuous variables. In addition, two logistic regression analyses were conducted. The first had ADP in the past year as the outcome. The second had PMH as the outcome.

In the subsample of those with a history of cannabis dependence (*n* = 336), three logistic regression analyses were conducted. The first had freedom from cannabis dependence in the past year as the outcome. The second had freedom from mental illness, suicidal thoughts, and cannabis and other drug or alcohol dependence in the past year as the outcome. The third had PMH as the outcome. The included variables were all chosen a priori based upon the published literature and were entered in simultaneously.

Sample sizes are reported in their original, unweighted form, but all data were weighted to adjust for the probability of selection and nonresponse.

## 3. Results

Among those with a history of cannabis dependence, 72% were in remission in the year preceding the survey. More than half (53%) of those with a history of cannabis dependence were free of any mental illness, suicidal thoughts, or addictions of any kind in the year preceding the survey, but this was dramatically lower than the 92% of those without a history of cannabis dependence who were free of all these disorders (*p* < .001) (Please see Table 1). Approximately two in every five (43%) respondents with a history of cannabis dependence were in PMH, which was considerably lower than the 74% of those without a history of cannabis dependence who were in PMH (*p* < .001). Those with a history of cannabis dependence were much more likely than those without to be male (71% vs. 49%), white (87% vs. 77%), to have ever had a major depressive episode (35% vs. 11%) or to have ever had generalized anxiety disorders (GAD) (27% vs. 9%). Adults with a history of cannabis dependence were much less likely to have completed a post secondary degree (47% vs. 65%) or to be married (36% vs. 66%). Those with a history of cannabis dependence were younger, had lower income and social support, and had a higher number of adverse childhood experiences.

Two sets of logistic regression analyses using the full sample (*n* = 20, 777) are provided in Table 2, the first set examines factors associated with the absence of psychiatric disorders (APD) in the preceding year and the second set has positive mental health (PMH) as the outcome. Those without a history of cannabis dependence had nine times the odds of APD in comparison to those who had ever been cannabis dependent (OR = 9.66; 95% CI = 7.75, 12.04). After taking into account socio-demographics, social support, and history of childhood adversities, and mental health problems, these odds declined but remained statistically significant (OR = 4.05; 95% CI = 3.03, 5.41). In the second set of analyses, adults without a history of cannabis dependence had more than three times the odds of PMH compared to those who had been cannabis dependent at some point in their lives (OR = 3.81; 95% CI = 3.06, 4.75). These odds declined substantially after full adjustment (OR = 1.82; 95% CI = 1.40, 2.35).

We identified the factors associated with remission for at least the past year from cannabis dependence among the 336 respondents who had ever been dependent on cannabis (please see Table 3). Women had twice the odds of remission in comparison to men (OR = 2.24; 95% CI = 1.16, 4.35). Each decade of age doubled the odds of remission (OR = 2.26; 95% CI = 1.61, 3.17). Those who had never experienced major depressive disorder had almost double the odds of remission in comparison to those with a history of depression (OR = 1.90; 95% CI = 1.09, 3.33). For each point higher on the social provision scale, a measure of social support, the odds of remission increased by 9% (OR = 1.09; 95% CI = 1.02, 1.16). White respondents had approximately half the odds of remission compared to nonwhite; however, this variable failed to reach statistical significance with a *p*-value of 0.06. The following variables were not statistically significant in this logistic regression analysis: education level, income, number of adverse childhood experiences, marital status, and history of GAD.

Table 3 also provides the results of the logistic regression analysis examining factors associated with the absence of psychiatric disorders (APD) in the past year in those with a history of cannabis dependence. Psychiatric disorders encompassed suicidality, addictions to alcohol and/or drugs, including cannabis, major depressive disorders, generalized anxiety disorders, and bipolar disorders. The odds of APD were double for women as opposed to men (OR = 2.56; 95% CI = 1.36, 4.85), and for those who had never experienced either depressive disorders or anxiety disorders in their life in comparison to those with such experiences (depression OR = 2.87; 95% CI = 1.64, 5.04; GAD OR = 2.42; 95% CI = 1.28; 4.60). With each decade of age, the odds of APD doubled (OR = 2.14; 95% CI = 1.58, 2.91). With every additional point on the social provision scale the odds of APD increased by 22% (OR = 1.22; 95% CI = 1.13, 1.31). The following factors were not statistically significant in the logistic regression analysis: race, education, income, and number of adverse childhood experiences.

Table 3 also reports on the logistic regression analysis of factors associated with PMH among those with a history of cannabis dependence. Women had more than twice the odds of PMH in comparison to men (OR = 2.55; 1.33, 4.90). With each decade of age, the odds of PMH more than doubled (OR = 2.36; 95% CI = 1.72, 3.23). With each additional point on the social provision scale, the odds of PMH increased by 34% (OR = 1.34; 95% CI = 1.22, 1.48). Individuals who had never experienced GAD had triple the odds of PMH in comparison to those who had ever had an anxiety disorder (OR = 3.41; 95% CI = 1.70, 6.83). There was a trend towards statistical significance (*p* = 0.08) in adults without a history of major depressive disorder. They had higher odds of PMH than those who had experienced major depression (OR = 1.70; 95% CI = 0.94, 3.08). When employing all variables simultaneously in this logistic regression analysis, neither race nor education level nor income nor number of childhood adversities nor marital status reached statistical significance.

## 5. Discussion

This nationally representative Canadian study indicates that almost three quarters of individuals who had ever been addicted to cannabis at some point in their lives are in remission in the past year. However, we cannot determine from these data the duration of that remission, as many individuals may relapse back into dependence. Furthermore, we certainly do not want to suggest that these findings in any way negate the well-established negative long-term consequences of cannabis dependence on educational and occupational outcomes and other negative sequelae.

It is important to note that adults without a history of cannabis dependence had more than nine times the odds of APD and more than three times of the odds of PMH in comparison to those with a history of cannabis dependence. Even after adjusting for a wide range of socio-demographic characteristics, social support, early traumas and history of mental illness, those without a history of cannabis dependence still had significantly higher odds of APD and PMH than those who had ever been addicted to cannabis. These results suggest a strong association between history of cannabis dependence and problematic mental health outcomes.

Our results identified a number of factors that were associated with remission that were also consistent with the literature. Women were more likely to be in remission, to be free of mental illness (APD) and have positive mental health. It is possible that women may have more acutely negative physical, mental, and social consequences of substance use than do men [15, 32]. This may encourage them to pursue strategies to overcome cannabis addiction. Women may also decrease substance use during pregnancy or periods of child-rearing due to side effects and associated feelings of guilt [33]. It is perplexing that we found, as had Lopez-Quintero Catalina and colleagues [15], that marriage did not play a role in remission.

With each decade of age, an individual's odds of remission doubled. Decreases in impulsivity, increased role responsibility, awareness of the impact of drug use on health as well as the social and legal consequences are thought to play a role in remission among older individuals [15, 17, 19]. 

Recovery from substance use goes beyond mere abstinence and includes a state of improved physical and psychological health, wellness, and quality of life [25, see reference list below]. We suggest that PMH is a promising measure of optimal recovery in that it encompasses freedom from all substance dependence, mental illness, and suicidality in addition to almost daily happiness and/or life satisfaction and social and psychological well-being.

When considering predictors of remission from cannabis dependence and achievement of PMH, it is important to also examine factors that may play a role in lifetime history of depression and anxiety. Lifetime history of depression and/or anxiety was strongly associated with each of the three outcomes we examined in this study. Individuals without a history of depression had almost double the odds of remission. It is possible that individuals who are depressed have a harder time motivating themselves to abstain from drug and alcohol use as they may use substances to improve mood through self-medication [34]. Those without a history of depression and anxiety had twice the odds of also being free of APD. Furthermore, those without a history of anxiety had triple the odds of PMH compared to those who had ever experienced an anxiety disorder. Depression was negatively associated with cannabis remission and APD, and although suggestive, the association between lifetime depression and PMH did not reach statistical significance (*p* = 0.08) when anxiety disorders were also included in the analysis. The reasons for the comorbidity between cannabis use and lifetime history of psychiatric disorders remain unclear, although, some research suggests that cannabis use may influence the initiation and of progression of psychiatric disorders [21]. Additionally, it is possible that cannabis use may share common etiologic pathways with certain psychiatric disorders and/or cannabis may also be used to manage or improve pre-existing psychiatric symptoms [21]. These findings underline the importance of interventions to address mental illness, as it appears to be a key barrier to remission and recovery from cannabis dependence.

Social support played a significant role in not only achieving remission from cannabis, but it was also a strong predictor of APD and PMH. With every additional point on the social provision scale the odds of remission increased by 9%, the odds of APD increased by 22% and the odds of PMH increased by 34%. This highlights the significance of access to strong social supports for individuals who are on the road to recovery. Our results about the robust impact of social support are consistent among the literature. For example, Kaskutas et al. [35] identify relationships and supportive social networks as essential for any recovery. If strong social support helps individuals reach full recovery, it is important to consider ways to best facilitate social integration and social support for clients who are recovering from cannabis addiction. Clinicians may be more effective if they expand their focus on treatment for cannabis to include strategies to assist clients in creating and maintaining social connectedness. Fostering and utilizing social capital may aid in the recovery from substance dependence.

Unfortunately, the CCHS-MH did not contain information on what interventions, if any, the respondents with a history of cannabis dependence had accessed to assist them with quitting cannabis. A recent systematic review of randomized controlled trials of psychosocial interventions for cannabis cessation suggests that clinicians have several promising interventions to draw upon in order to effectively treat their patients with cannabis abuse and/or dependence in order to promote remission and recovery [36]. Efficacious treatment options include cognitive behavioural therapy and motivational interviewing [36]. Combined treatments such as motivational enhancement therapy and cognitive behavioural therapy show even greater success in reducing cannabis use as well as dependence related symptoms compared to delivering these therapies individually [37]. Results of the current study may be useful to inform future evidence-based treatments by our identification of modifiable variables associated with PMH such as increasing social support and addressing targeted outreach to those who are least likely to experience remission including younger individuals, men, and those with a history of depression.

There are several limitations in the current study. First, as publicly available data were used, only information that had been gathered in the original survey could be analyzed. For example, we were unable to determine potentially important factors such as the age of onset of cannabis use, the duration of dependence and the age at remission. Additionally, there was no information whether the respondent had used any interventions to assist with the remission process and if so, what interventions they used. Furthermore, previous research indicates that individuals who have comorbid cocaine dependence have 90% lower odds of CUD remission [15]. Future research should investigate different trajectories of recovery among those with comorbid cocaine and other substance dependence compared to those without any other forms of substance dependence.

Second, as we were limited to cross-sectional data, we could not determine the sequence of events nor a causal pathway. For example, it is impossible to determine the onset or duration of depression and generalized anxiety disorders and whether these factors preceded cannabis dependence or were a result of the addiction. A longitudinal study that follows individuals addicted to cannabis over a long period of time is necessary for better understanding of the process by which remission from cannabis occurs and through which positive mental health is achieved. Future longitudinal research would benefit from more exact information on the timing and duration of cannabis use, interventions used, as well as a thorough investigation of the potential role of these factors in resilience and recovery from cannabis dependence.

Third, the retrospective and self-report nature of this study are other limitations that merit further discussion. Several studies have suggested that reports of psychological disorders and mental illness are subject to recall and social desirability biases [38]. The implications of these biases could mean that lifetime cannabis dependence may be underreported and the prevalence of remission may also be inaccurate. Future studies should use more objective measures to determine lifetime cannabis dependence in order to avoid these problems. Furthermore, assessment of childhood maltreatment would be improved if the respondents' child welfare records were used.

Fourth, the response rate for the CCHS-MH was slightly under 70%, which raises the question of whether individuals who were not in “positive mental health” were less likely to complete the survey, thereby biasing our sample towards higher rates of recovery. Another limitation that warrants consideration is the lack of information available on individuals under the age of 20. Although cannabis use among adolescence in Canada has substantially declined since 2008/09 [39], use of the substance is particularly high with Canadian youth having one of the highest rates of cannabis use among industrialized countries [40]. Additionally, the social and legal landscape surrounding cannabis use has recently changed in Canada due to legalization of cannabis [41]. This retrospective study does not capture PMH among those under age 20, who may be particularly vulnerable to negative long-term outcomes and future research should include current information on cannabis use trends among youth and younger adult populations, particularly in the context of the recent legalization.

This study did not account for individuals who consume cannabis at a frequency, intensity, or duration not meeting DSM-criteria for substance dependence. Since it only included individuals who were dependent on cannabis, results cannot be generalized to the much larger number of Canadians who use cannabis but are not dependent upon it. Lastly, the sample size of those with a history of cannabis dependence (*n* = 336) was relatively small so that the power was less than ideal. Future studies would benefit from larger sample sizes.

Despite these limitations, this study identifies factors associated with cannabis remission, the absence of psychiatric disorders or addictions in the past year, and positive mental health in a nationally representative sample of Canadians with a history of cannabis dependence. These findings are particularly valuable to inform future outreach, targeting, and interventions in light of the increasing number of cannabis users expected as a result of recent legalization, and the large number of individuals dependent on cannabis.

## Figures and Tables

**Table 1:** Characteristics of those with and without a history of cannabis dependence (*n* = 20, 777).

	No history of cannabis dependence	With history of cannabis dependence	*p*-value
	(*n* = 20, 441)	(*n* = 336)	
	*N* (%)	*N* (%)	
	Mean ± SD	Mean ± SD	
*Current cannabis dependence*			
Yes	0 (0.0%)	93 (27.8%)	<0.001
No	20,443 (100.0 %)	241 (72.2%)	
*Positive mental health*			
No	5,337 (26.1%)	192 (57.5%)	<0.001
Yes	15,106 (73.9%)	142 (42.5%)	
*Current mental illness*			
1 or more mental illnesses	1,719 (8.4%)	157 (47.0%)	<0.001
None	18,724 (91.6%)	177 (53.0%)	
*Sex*			
Male	9,977 (48.8%)	238 (71.3%)	<0.001
Female	10,466 (51.2%)	96 (28.7%)	
*Race/ethnicity*			
White	15,823 (77.4%)	290 (86.8%)	<0.001
Visible minority	4,620 (22.6%)	44 (13.2%)	
*Age*	47.80 ± 16.78	34.42 ± 11.20	<0.001
*Education*			
No post-secondary degree	7,227 (35.4%)	177 (53.2%)	<0.001
Post-secondary degree	13,216 (64.6%)	156 (46.8%)	
*Household income (in thousands of dollars)*	51.55 ± 28.54	45.88 ± 29.74	<0.001
*Adverse childhood events*	0.36 ± 0.63	0.90 ± 0.94	<0.001
*Marital status*			
Single/divorced/widowed	6,893 (33.7%)	214 (64.1%)	<0.001
Married/common-law	13,550 (66.3%)	120 (35.9%)	
*Social provisions scale*	36.05 ± 4.33	35.29 ± 4.80	0.002
*Lifetime history of major depressive disorder*			
Yes	2,256 (11.0%)	118 (35.3%)	<0.001
No	18,188 (89.0%)	216 (64.7%)	
*Lifetime history of generalized anxiety disorder*			
Yes	1,759 (8.6%)	90 (26.9%)	<0.001
No	18,684 (91.4%)	244 (73.1%)	

**Table 2:** Logistic regression analyses predicting freedom from past-year mental illness and positive mental health (*n* = 20, 777).

	Outcome: no past-year substance dependence or mental illness		Outcome: positive mental health	
	Block 1	Block 2	Block 1	Block 2
	OR (95% CI)	OR (95% CI)	OR (95% CI)	OR (95% CI)
*Lifetime cannabis dependence*				
Yes (ref.)	1.00	1.00	1.00	1.00
No	9.66 (7.75, 12.04)	4.05 (3.03, 5.41)	3.81 (3.06, 4.75)	1.82 (1.40, 2.35)
*Sex*				
Male (ref.)	—	1.00	—	1.00
Female	—	1.27 (1.12, 1.43)	—	1.08 (1.01, 1.16)
*Race/ethnicity*				
White	—	0.80 (0.69, 0.93)	—	0.88 (0.81, 0.96)
Visible minority (ref.)	—	1.00	—	1.00
*Age*	—	1.39 (1.34, 1.45)	—	1.19 (1.17, 1.22)
*Education*				
No post-secondary degree (ref.)	—	1.00	—	1.00
Post-secondary degree	—	0.91 (0.80, 1.04)	—	0.95 (0.88, 1.02)
*Household income*	—	1.00 (1.00, 1.01)	—	1.00 (1.00, 1.00)
*Adverse childhood events*	—	0.71 (0.66, 0.77)	—	0.75 (0.71, 0.79)
*Marital status*				
Single/divorced/widowed (ref.)	—	1.00	—	1.00
Married/common-law	—	1.44 (1.27, 1.63)	—	1.23 (1.14, 1.33)
*Social provisions scale*	—	1.12 (1.10, 1.13)	—	1.18 (1.17, 1.19)
*Lifetime history of major depressive disorder*				
Yes (ref.)	—	1.00	—	1.00
No	—	10.69 (9.43, 12.13)	—	3.69 (3.32, 4.10)
*Lifetime history of generalized anxiety disorder*				
Yes (ref.)	—	1.00	—	1.00
No	—	5.29 (4.59, 6.09)	—	2.92 (2.60, 3.28)
*−2 Log likelihood*	12265	8228.6	23931	19936
*Nagelkerke R square*	0.035	0.42	0.01	0.26

**Table 3:** Logistic regression analyses predicting (1) freedom from past-year cannabis dependence, (2) no substance dependence or past- mental illness, (3) positive mental health in a sample of individuals with history of cannabis dependence (*n* = 336).

	No past-year cannabis dependence		No past-year substance dependence or mental illness		Positive mental health	
	OR	95% CI	OR	95% CI	OR	95% CI
*Sex*						
Male (ref.)	1.00	REF	1.00	REF	1.00	REF
Female	2.24	1.16, 4.35	2.56	1.36, 4.85	2.55	1.33, 4.90
*Race/ethnicity*						
White	0.45	0.20, 1.05	0.53	0.25, 1.16	0.75	0.34, 1.69
Visible minority (ref.)	1.00	REF	1.00	REF	1.00	REF
*Age*	2.26	1.61, 3.17	2.14	1.58, 2.91	2.36	1.72, 3.23
*Education*						
No post-secondary degree (ref.)	1.00	REF	1.00	REF	1.00	REF
Post-secondary degree	1.42	0.82, 2.44	0.94	0.56, 1.60	0.86	0.49, 1.49
*Household income*	1.00	0.99, 1.01	1.00	0.99, 1.01	1.00	0.99, 1.01
*Adverse childhood events*	0.90	0.62, 1.30	1.07	0.75, 1.52	1.28	0.88, 1.84
*Marital status*						
Single/divorced/widowed (ref.)	1.00	REF	1.00	REF	1.00	REF
Married/common-law	0.77	0.44, 1.35	1.29	0.76, 2.21	1.21	0.70, 2.10
*Social provisions scale*	1.09	1.02, 1.16	1.22	1.13, 1.31	1.34	1.22, 1.48
*Lifetime history of major depressive disorder*						
Yes (ref.)	1.00	REF	1.00	REF	1.00	REF
No	1.90	1.09, 3.33	2.87	1.64, 5.04	1.70	0.94, 3.08
*Lifetime history of generalized anxiety disorder*						
Yes (ref.)	1.00	REF	1.00	REF	1.00	REF
No	1.28	0.67, 2.44	2.42	1.28, 4.60	3.41	1.70, 6.83

*−2 Log likelihood*	346.2		369.4		348.2	
*Nagelkerke R square*	0.20		0.33		0.38	

## Data Availability

This analysis uses secondary analysis of a publicly available data set: Statistics Canada's 2012 Canadian Community Health Survey- Mental Health.
